# Research on the impact of restorative environmental perception on tourists’ environmental responsibility behavior in mountain-type scenic spots: taking Taishan Scenic Spot as an example

**DOI:** 10.3389/fpsyg.2026.1742474

**Published:** 2026-02-25

**Authors:** Pengfei Tai, Fugao Jiang, Zhuang Zhuang, Linliang Zou, Xinchun Wang

**Affiliations:** 1 School of Physical Education and Sport Science, Qufu Normal University, Jining, China; 2 College of Physical Education and Science, East China Normal University, Shanghai, China

**Keywords:** environmental experience, environmentally responsible behavior, exercise experience, restorative environmental perception, Taishan Scenic Area, visitor satisfaction

## Abstract

**Introduction:**

The environmental quality and sustainable development of mountain-type scenic spots are profoundly influenced by the behaviors of visitors. Tourists’ environmentally responsible behavior is crucial for maintaining this quality, yet the mechanisms linking it to restorative environmental perception, particularly within the context of sports tourism, require further exploration. This study, grounded in Attention Restoration Theory, investigates how tourists’ sports and environmental experiences in a mountain setting influence their environmentally responsible behavior, with a focus on the mediation effect of restorative environmental perception and satisfaction.

**Methods:**

This study adopted a quantitative research approach, utilizing an on-site survey questionnaire to empirically investigate the proposed model. It evaluated tourists’ exercise experience, environmental experience, the four dimensions of restorative environmental perception (being away, extent, fascination, compatibility), satisfaction, and environmentally responsible behavior. Data were collected via on-site interviews from 233 tourists who had completed their hiking visit. The data analysis employed structural equation modeling using Amos 24.0 to test the hypothesized relationships and the SPSS PROCESS macro for mediation analysis, using bootstrapping to confirm indirect effects.

**Results:**

The findings indicate that both exercise experience and environmental experience positively influenced tourists’ perceived restorativeness, with environmental experience demonstrating a stronger overall effect. Specifically, environmental experience significantly and positively affected all four dimensions (being away: β = 0.553, extent: β = 0.854, fascination: β = 0.919, compatibility: β = 0.809), while exercise experience only positively influenced the “being away” dimension (β = 0.351). Furthermore, the “extent” (β = 0.372) and “compatibility” (β = 0.449) dimensions of perceived restorativeness, along with both experience types, significantly enhanced tourist satisfaction. Satisfaction, in turn, was a strong positive predictor of environmentally responsible behavior (β = 0.728). Mediation analysis confirmed that satisfaction fully mediated the relationship between “being away” and environmentally responsible behavior, and partially mediated the relationships for the other three dimensions. A significant chain mediation effect was also supported, revealing that perceived restorativeness and satisfaction sequentially transmit the influence of exercise experience onto environmentally responsible behavior.

**Conclusion:**

For mountain-type scenic spots aiming to promote environmentally responsible behavior, enhancing both the sports and environmental experiences for tourists is paramount. By improving these experiences, destinations can foster a stronger sense of restorative perception and increase visitor satisfaction, which subsequently encourages pro-environmental actions. This study provides a validated theoretical model and practical insights for the sustainable management and market-specific strategy development of mountain tourism destinations. Moreover, the study reveals differential effects among restorative dimensions, providing nuanced insights into their distinct roles in the psychological restoration–behavior chain.

## 1 Introduction

Mountaineering tourism, as one of the closest forms of human-nature contact, is an important way to promote the transformation of ecological value, and an important part of China’s ecological civilization construction, as of the end of 2024, the number of outdoor sports participants in China has exceeded 400 million (Zhou et al., 2025), the outdoor sports market, including mountaineering, has entered into a stage of rapid development, but with the increase in the number of tourists, the mountaineering process with the increase of tourists’ uncivilized behavior has caused damage to the environment of mountain-type scenic spots, seriously affecting the attractiveness and ecological health of tourist destinations. Tourists as the main body of the tourism process and the primary implementer of environmental behavior, the study of environmentally responsible behaviors is of great significance to reduce the negative impacts of tourists’ recreational process on the environment and to improve the sustainable development capacity and management capacity of scenic spots (Rodríguez-Oromendía et al., 2013). Based on this, guiding tourists to voluntarily make behaviors conducive to environmental protection in scenic spots has become an increasing focus and hotspot in society and academia. Exploring the influence mechanism between restorative perception and environmentally responsible behaviors can help to provide theoretical support and value guidance for improving tourists’ ecological awareness to promote the sustainable development of scenic spots.

Currently, academic research primarily investigates the relationship between restorative environmental perception, exercise experience, environmental experience, visitor satisfaction, and environmentally responsible behavior through four main thematic clusters: definition and dimensional measurement, theoretical formation mechanisms, contextual moderators, and integrated experience models. (1) Regarding definition and dimensional measurement, scholars have defined restorative environmental perception based on attention restoration theory, encompassing dimensions such as “being away” “compatibility” “fascination” and “extent” (Cho and Um, 2016). For environmentally responsible behavior, definitions have evolved from generic traits to multi-dimensional structures, distinguishing between general and site-specific behaviors (Lee and Jan, 2013). In the context of exercise experience, subjective scales such as the subjective exercise experience scale have been developed to measure positive wellbeing, while environmental experience is often conceptualized through the 4E theory (Entertainment, Education, Aesthetics, Escapism) (Weng et al., 2023). (2) Regarding theoretical formation mechanisms, extensive research utilizes frameworks like the theory of planned behavior (Xie et al., 2022), norm activation theory (Xu et al., 2019), and the Stimulus-Organism-Response model (Su and Swanson, 2020) to explain how environmental perception and experience lead to ERB. Studies confirm that environmental knowledge, environmental sensitivity, and place attachment are critical mediators in this process (Lee, 2015; Cheng et al., 2022). The Stimulus-Organism-Response model is particularly prevalent, illustrating how restorative perceptions evoke emotional states such as awe or satisfaction, which in turn trigger environmentally responsible behavior (Jiang and Gao, 2022; Xu and Hu, 2024). (3) In terms of contextual moderators, literature highlights that the link between perception and behavior is not universal but is conditioned by factors such as social class (He et al., 2026), demographic characteristics (Tseng et al., 2024), and social interactions (Tu and Ma, 2022). For instance, negative message framing has been found to be more persuasive for lower-class tourists, whereas positive framing is more effective for upper-class tourists (He et al., 2026). Additionally, the level of environmental commitment and cultural background (Chen and Liu, 2025) significantly moderates the effectiveness of psychological drivers on environmentally responsible behavior. (4) Concerning integrated experience models, recent studies increasingly focus on the interplay of multiple experiential factors. Research indicates that serious leisure and creative tourism experiences (Lu et al., 2025) positively influence ERB by enhancing destination attractiveness and place attachment (Liu et al., 2024). Moreover, the integration of auditory and visual landscapes has been shown to significantly impact visitor satisfaction and restorative perception (Li et al., 2022; Zhu et al., 2024). The use of emerging technologies like Virtual Reality to induce “ecological presence” has also been identified as a novel pathway to foster environmentally responsible behavior (Su et al., 2024), highlighting the expanding scope of experience research. Overall, although the academic community has conducted rich interdisciplinary research on the conceptualization, measurement, and driving factors of environmentally responsible behavior, existing studies mostly examine these constructs in isolation or treat restorative perception as a unidimensional antecedent variable. Particularly lacking is an integrated sequential model that systematically examines how exercise experience and environmental experience in mountain sports tourism influence environmentally responsible behavior through the multidimensional mechanisms of restorative perception, ultimately mediated by satisfaction.

Taishan Scenic Area runs through the middle of Shandong Province, with high endowment of sports tourism resources, diversified types of products, and vast space for development. Specifically manifested as: rich natural reserves, with a variety of ecological environments, peculiar natural landscapes and abundant mountain springs and streams; strong humanistic heritage, with a long history, rich cultural heritage and long folk beliefs; significant support for the regional economy, with a variety of industrial ecosystems, a diversified industrial structure, a strong power of development; superior potential for the development of sports tourism, with a faithful mountaineering The development potential of sports tourism is superior, with loyal mountaineering market groups, diversified industrial structure and diversified mountaineering tourism modes. As a world cultural and natural double heritage and national 5A level scenic spot, it has rich tourism resources, among which the four major wonders of “the rising sun, the sunset sun, the Taishan Buddha light, the sea of clouds and the jade disk” are the most famous, forming the core viewing competitiveness that distinguishes it from other types of scenic spots. As a representative and exemplary mountain-type scenic area.

As an important carrier of ecological civilization construction and great health cause, there are few studies on the relationship between tourists’ exercise experience, restorative environmental perception and environmental responsibility behaviors in the context of mountain-type scenic spots, which is inconsistent with the important position of mountain-type scenic spots in the promotion of ecological construction and the cause of great health. The present study adopts the classical structural equation model and relies on the theory of attention restoration to the World Natural Heritage and World Cultural Heritage tourist destination Taishan Mountain Scenic Area, which is a world natural heritage and world cultural heritage tourist destination. World Natural Heritage and World Cultural Heritage tourist destination Taishan Scenic Spot as an empirical case, to reveal the influence mechanism of restorative environmental perception on environmental responsibility behavior of tourists in mountain-type scenic spots, with the hope of further enriching the research results on the drivers of tourists’ restorative environmental perception and environmental responsibility behavior, and providing a mirror for the realization of the high-quality development of mountain-type scenic spots.

## 2 Research hypotheses and model proposal

### 2.1 Conceptual foundations and definitions

This study is grounded primarily in Attention Restoration Theory (ART) (Kaplan, 2001; Berman et al., 2009), which posits that natural environments possess specific restorative qualities that can facilitate the recovery of directed attention and reduce mental fatigue. The theoretical model also incorporates insights from affective evaluation perspectives, where experiences and perceptions are posited to influence satisfaction and subsequent behavioral intentions (Liu and Jang, 2009). To ensure conceptual clarity, the key constructs employed in this study are defined as follows:

#### 2.1.1 Restorative environmental perception

Following Attention Restoration Theory, this construct refers to an individual’s subjective experience that an environment facilitates the recovery of cognitive resources and provides a sense of psychological restoration. In this study, it is conceptualized as a higher-order construct comprising four distinct dimensions: being away, extent, fascination, and compatibility.

Being away refers to the psychological experience of feeling distant from one’s usual routines, demands, and sources of stress, achieved by being in a different physical or conceptual setting; Extent describes the richness and coherence of an environment, where the setting is perceived as a whole, ordered world that is large enough for the mind to explore freely; Fascination involves a form of attention that is captured effortlessly by inherently interesting features in the environment, requiring no directed effort and thereby allowing fatigued attentional systems to rest; Compatibility refers to the degree of fit between an individual’s purposes, inclinations, or tendencies and what the environment supports, demands, or affords, enabling desired activities to be carried out with ease.

#### 2.1.2 Exercise experience

This construct denotes a tourist’s holistic subjective evaluation of the physical activity undertaken during the visit. It encompasses perceptions of safety, enjoyment, social interaction during the activity, and the sense of physical and mental reward derived from it [adapted from Jang and Kim (2022)].

#### 2.1.3 Environmental experience

This construct refers to a tourist’s overall subjective assessment of the destination’s natural and man-made attributes, including the quality of natural resources, the comfort of the ecological setting, the adequacy of facilities and services, and the appeal of cultural and historical elements (Giovanna et al., 2017).

#### 2.1.4 Visitor satisfaction

This is defined as the affective state resulting from a tourist’s post-consumption evaluation, where the actual experience of the destination is judged against prior expectations, leading to an overall feeling of pleasure or fulfillment (Rojas and Camarero, 2008).

#### 2.1.5 Environmentally responsible behavior

This construct pertains to the specific actions and commitments undertaken by tourists during their visit, aimed at minimizing negative impacts on the local environment or actively contributing to its preservation and sustainable development (Jiang and Gao, 2022).

### 2.2 Arousal of tourists’ restorative perception

#### 2.2.1 Tourists’ exercise experience and restorative perception

When individuals participate in sports activities, it can improve the individual’s sense of wellbeing and satisfy the individual’s health needs, and when tourists exercise in scenic spots, a number of physiological indicators will change, which will significantly improve their physiological and psychological conditions (Kaplan, 2001), the psychological restoration of tourists is affected by the greater pattern of tourists’ exercise behavior, while the quality of the tourists’ exercise experience also affects the psychological restoration of tourists, such as the mountaineering process of fun and safety will all affect tourists’ exercise experience, which in turn will have an effect on the restorative perception, so the following hypotheses are proposed:

H1: Tourists’ exercise experience positively affects tourists’ perceived restorativeness.

H1a: Tourists’ exercise experience positively affects tourists’ perceived restorativeness of bing away.

H1b: Tourist movement experience positively influences tourists’ perceptions of environmental restorativeness in extent.

H1c: Tourist movement experience positively influences tourists’ perceptions of environmental restorativeness as fascination.

H1d: Tourist movement experience positively influences the compatibility of restorative perceptions of tourist environment.

#### 2.2.2 Tourist environmental experience and restorative perception

When individuals leave the original social environment and come to the natural environment, they can relieve mental stress and negative affect through the natural environment to achieve the effect of energy recovery (Cui et al., 2025). In turn, tourists’ environmental restorative perception is closely related to the quality of the natural environment (Zhai et al., 2025), and even green spaces in dense buildings in urban areas can have a positive impact on individuals’ restorative perception (Li et al., 2023).

Based on this, the inference is proposed that tourists’ environmental experience has a significant effect on tourists’ environmental restorative perception, and when tourists have a better experience and affect of the environment, the psychological restoration of the environment will be more obvious to tourists, and tourists’ mental state will get a better relaxation effect, so the hypothesis is proposed:

H2: Scenic environment experience positively affects tourists’ environmental restorative perception.

H2a: The environmental experience of the scenic area has a positive impact on “bing away.”

H2b: Scenic environment experience positively affects tourists’ environmental restorative perception of extent.

H2c: Scenic environment experience positively influences tourists’ perceived restorativeness of fascination.

H2d: the compatibility of scenic environment experience positively influencing tourists’ perceived restorativeness.

### 2.3 Tourist restorative perception and satisfaction

Among the current studies, there are relatively more studies on the structural dimensions of tourists’ perceived restorativeness, and some of them believe that tourists’ restorative perceptions deeply affect tourists’ affect and satisfaction, for example, restorative perceptions of zoo tourists at the end of their visit will have a certain impact on tourists’ pleasure and place preference (Li et al., 2023), while tourists, when they are in places such as cultural heritage tourism sites and national parks, the Tourist environmental satisfaction will have a certain impact on tourists’ satisfaction (Fan et al., 2014), when tourists carry out sports tourism activities and get restorative perception experience, they will get restorative physically and mentally, and their ideology gradually tends to be calm, when tourists’ perceived restorativeness are better, their bodies get better psychological restoration, and the evaluation of the trip will be higher, which will result in a higher satisfaction and contentment, therefore, it is proposed to hypothesize that:

H3a–H3d: Bing away, extent, fascination, and compatibility positively influence satisfaction.

### 2.4 Tourist EXPERIENCE AND SATISFACTION

Satisfaction in the field of tourism mainly refers to when tourists after visiting the destination, the actual experience of the process to produce the evaluation, and the factors affecting tourist satisfaction is multi-faceted, part of the research focuses on from the perspective of the tourists’ perceived value, through the way of empirical research to argue that the tourists’ perceived value will bring a positive impact on the satisfaction of the tourists’ travel (Tian et al., 2015). Tourists’ perceived value in terms of tourism cognition affects tourists’ satisfaction, and the source of tourists’ satisfaction is related to the cognition and affect of the tourism product, which comes from the consumption experience in the process of carrying out tourism consumption (Zhang Q. et al., 2020). It can be seen that the affect and perceptions of tourists are prerequisites and key antecedent variables for tourists to generate satisfaction. Therefore the hypothesis is proposed:

H4: Tourist exercise experience positively affects tourist satisfaction.

H5: Tourists’ environmental experience positively affects tourists’ satisfaction.

### 2.5 Mediation effect of environmentally responsible behavior and satisfaction

Tourist environmentally responsible behavior generally refers to a series of means and methods implemented by tourists in the process of tourism activities in tourist destinations that are conducive to environmental protection and sustainable development of the destination, and also refers to actions taken by tourists in order to reduce or avoid any negative impacts on the local environment during their stay in the tourist destination. Among the objective and subjective factors that affect tourists’ environmental responsibility behavior, the objective factors are mainly related to the environmental quality of the destination itself (Zhou et al., 2025), the relevant laws and regulations of the tourism destination regarding the environment (Barreal et al., 2024), and the destination’s education for tourists (Zhang T. et al., 2020); Subjective individual factors are mainly related to tourists’ cognitive factors (Liu and Wang, 2020) and tourists’ affect factors (Wang and Tou, 2021). In recent studies, scholars have demonstrated that the level of tourist satisfaction affects their environmental responsibility behavior in forest park tourism (Lonnstedt, 2012), ecotourism (Rosmadi et al., 2024), and heritage tourism (Dogan et al., 2019). Although the research backgrounds are different, most of the evidence confirms that tourist satisfaction has a direct positive impact on tourist environmental responsibility behavior, and the mediation effect of satisfaction has also been verified (Patwary, 2023). Therefore, a hypothesis is proposed:

H6: Tourist satisfaction positively affects environmentally responsible behavior.

H7: Tourist satisfaction plays a mediation effect in the effect of tourists’ perceived environmental restorativeness on their environmentally responsible behavior.

H7a: Tourist satisfaction mediates the effect of tourists’ perceived extensiveness of environmental restorativeness on their environmentally responsible behavior.

H7b: Tourist satisfaction plays a mediation effect in the effect of tourists’ perceived restorativeness of environmental restorativeness on their environmentally responsible behaviors.

H7c: Tourist satisfaction plays a mediation effect in the effect of tourists’ perceived environmental restorative fascination on their environmentally responsible behaviors.

H7d: Tourist satisfaction plays a mediation effect in the effect of tourists’ perceived restorativeness of compatibility on their environmentally responsible behavior.

### 2.6 Chain mediation effect of restorative perception and satisfaction

Based on the above analysis, tourists’ exercise experience and environmental experience positively affect restorative environmental perception, and the arousal of restorative environmental perception contributes to satisfaction, based on this it is hypothesized that restorative environmental perception and satisfaction may play a chain mediation effect between tourists’ exercise experience and environmentally responsible behaviors and the following hypotheses are proposed:

H8: Perceived restorativeness and satisfaction play a chain mediation effect in the influence of tourists’ exercise experience on their environmentally responsible behavior.

H8a: Distance and satisfaction play a chain mediation effect in the influence of tourists’ exercise experience on their environmentally responsible behavior.

H8b: Extent and satisfaction play a chain-mediated role in the influence of tourists’ exercise experience on their environmentally responsible behaviors.

H8c: Fascination and satisfaction play a chain-mediated role in the influence of tourists’ exercise experience on their environmentally responsible behaviors.

H8d: Compatibility and satisfaction play a chain mediation effect in the influence of tourists’ exercise experience on their environmentally responsible behavior.

H9: Perceived restorativeness and satisfaction play a chain mediation effect in the influence of tourists’ environmental experiences on their environmentally responsible behavior.

H9a: Bing away and satisfaction play chain-mediated roles in the influence of tourists’ environmental experience on their environmentally responsible behaviors.

H9b: Extent and satisfaction play a chain mediation effect in the influence of tourists’ environmental experience on their environmentally responsible behavior.

H9c: Chain mediation effect of fascination and satisfaction in the influence of tourists’ environmental experiences on their environmentally responsible behaviors.

H9d: Compatibility and satisfaction play a chain-mediated role in the influence of tourists’ environmental experience on their environmentally responsible behaviors.

## 3 Research design and data collection

### 3.1 Scale items design

**FIGURE 1:**
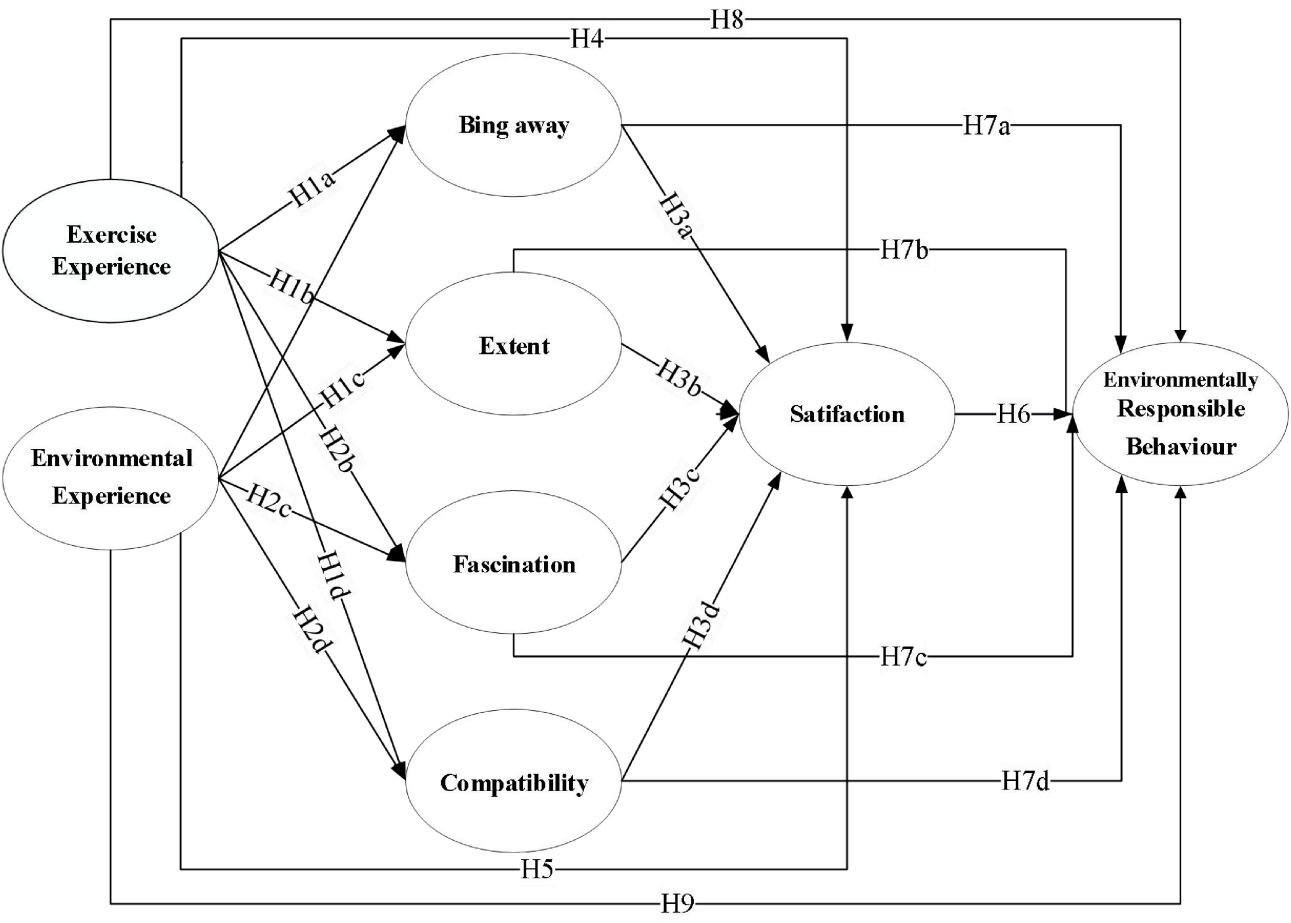
Proposed theoretical model.

This study draws on the research of Zhang and Liu (2021), Yoon and Uysal (2005), Chen and Tsai (2007), Chiu et al. (2014), Jia and Lin (2015), and synthesizes the PRS, RCS, PRCQ, and PDRQ scales, and appropriately adapts the scale items to the reality of the Taishan Scenic Area. The final scale items consists of six parts:

The first part is to investigate the demographic characteristics of tourists; the second part is used to investigate the exercise experience of tourists in the process of climbing Mount Tai with a total of 5 items; the third part is used to investigate the environmental experience of tourists with a total of 4 items; the fourth part is to investigate the restorative environmental perception of tourists in mountain scenic areas, which includes four dimensions with a total of 15 items; the fifth part is to investigate the situation of tourists’ satisfaction, which mainly includes 4 items; The sixth part is the survey of environmentally responsible behavior, which mainly includes 8 items (Figure 1); the second to sixth parts of which were measured using a five-point Likert scale, ranging from 1 (“Strongly Disagree”) to 5 (“Strongly Agree”). Higher scores indicate a stronger level of agreement with, or experience of, the measured construct.

### 3.2 Data collection and assessment of reliability and validity

In April 2025, at the exit of the Mount Tai scenic area, questionnaires were distributed to tourists who had completed the hiking experience using the random sampling method. To ensure the reliability of the questionnaire statistics, the team members were strictly trained to distribute and collect the questionnaires in the form of interviews and dialogues. To ensure research ethics, this study complied with the ethical guidelines of the Biomedical Ethics Committee of Qufu Normal University and the Belmont Report. Informed consent was obtained from all participants via written notices, and data were fully anonymized to protect participant confidentiality. After the statistics, a total of 260 questionnaires were recovered, excluding 27 questionnaires that were incomplete, incomplete answers and recognition of complete consistency, a total of 233 valid questionnaires were recovered, with a valid questionnaire rate of 90%, the questionnaire validity rate is high and the quality is good.

Using SPSS26.0 software to conduct exploratory factor analysis on 44 question items in the scale, the results show that from the above table, the KMO metric value is 0.914, which is greater than 0.8, the Bartlett’s test approximation chi-square value is 7,849.665, and the value of the degrees of freedom is 630, the *p*-value is 0.000 < 0.05, which achieves the level of significance, which indicates that the data is suitable for factor analysis.

The results indicated that Cronbach’s coefficient (Cronbach’s alpha) of 0.968 for the total scale of the questionnaire, and the reliability analyses of exercise experience, environmental experience, restorative environmental perception, satisfaction, and environmentally responsible behaviors yielded a result that the overall Cronbach’s coefficients (Cronbach’s alpha) of the scales were all greater than 0.8, indicating that the questionnaire has a high reliability level (Table 1).

**TABLE 1:** Validation factor analysis.

Latent variables	Observed variables	Factor loadings	Combined reliability CR	Average extracted variance AVE	Cronbach’s coefficient
Exercise experience	Exercise safety feasible	0.69	0.8489	0.53	0.87
	Interaction with peers is possible during exercise	0.766			
	Cognition are rewarding during climbing	0.776			
	Affect of satisfaction during exercise	0.744			
	High level of fun in sport	0.657			
Environmental experience	Rich in natural resources	0.857	0.9002	0.6939	0.899
	Good facilities and services	0.822			
	Comfortable ecological environment	0.901			
	Culture and History Attractive	0.744			
Bing away	Can get me out of my busy schedule	0.825	0.8802	0.6488	0.875
	Can keep me away from boring life	0.887			
	Can put me in a different place	0.726			
	Can give me complete rest and relaxation	0.775			
Extent midrule Attractiveness	The surrounding scenery is co-ordinated	0.737	0.871 0.831	0.6283> 0.625	0.802 0.871
	Quite curious about the unseen landscapes in Mount Tai	0.812			
	Makes me have significant good associations	0.821			
	The elements of the landscape are compatible Attractive qualities	0.798 0.853			
	Can entice me to explore and discover more	0.859			
	Would like to spend more time in Tarzan	0.64			
Compatibility	Everything here is in harmony with nature	0.681	0.8896	0.6704	0.886
	The excursion experience met my expectations	0.847			
	I was able to adapt quickly to the environment	0.884			
	I can do what I like	0.848			
Satisfaction	I was satisfied with my visit to Mount Tai compared to my expectations.	0.949	0.9617	0.8628	0.961
	Compared with similar places I have visited, the scenic spot gave me a better experience	0.911			
	I feel that the time, effort and money spent on visiting the Taishan Scenic Spot is worthwhile	0.929			
	Overall, I enjoyed my visit to Mount Tai very much	0.926			
Environmentally responsible behavior	I will dispose of the rubbish I produce while visiting	0.822	0.9262	0.6123	0.915
	I will comply with the environmental management standards of Taishan Scenic Spot when I visit.	0.817			
	I will take care of the plants, cultural relics and other tourism resources in the Taishan Scenic Area when I visit.	0.853			
	I will take care of the public services and tourism infrastructure in the Taishan Scenic Spot when I visit.	0.798			
	I think I have the responsibility to protect the ecological environment of Taishan Scenic Spot	0.79			
	I will take the initiative to adopt environmentally friendly behaviors during my visit.	0.703			
	I will remind others not to damage the environment when visiting	0.66			
	I will stop others from damaging the environment when visiting the city.	0.798			

Secondly, the validation factor analysis was established by Amos24.0 to assess the fitness of the model, and the data calculated by the software were compared with the standard fit indexes, and from the fit indexes obtained from the analysis, the chi-square/degree-of-freedom ratio was 2.921, which was in the range of 1–3, and Fang^[39]^ pointed out that the loosely specified value for chi-square degree-of-freedom ratio was 5, which proves that the model has a better fit. Rmsea value is 0.065.

After the above calculation and analysis, it can be seen that the model fit data constructed in this research meets the requirements. In addition, the factor loadings of each test item are greater than 0.6, the reliability of the combination of variables is higher than 0.8, and the AVE values are higher than the standard of 0.500, which indicates that the convergent validity of the model is good. Regarding the test of discriminant validity, there is a significant correlation between each test item (*p* < 0.01), and the absolute value of the correlation coefficient is less than 0.5, and all of them are less than the square root of the corresponding AVE, which indicates that there is a certain degree of correlation between the latent variables and a certain degree of differentiation between them, which indicates that the discriminant validity of the data of the scale is relatively satisfactory.

## 4 Data analysis and results

### 4.1 Basic demographic characteristics of respondents

According to the survey (Table 2), males accounted for 53% of the overall sample and females accounted for 47%, with the number of male tourists slightly higher than the number of female tourists; at the age level, young and middle-aged tourists aged 18–45 years old dominated, accounting for 73% of the total number of tourists; at the level of tourists’ education level, more than half of the respondents had bachelor’s degrees; at the occupation level, students, enterprise and public institution personnel and enterprise employees dominated; at the level of monthly income, tourists with a monthly income of 2001–5000 were the main target and enterprise employees; at the monthly income level, tourists with a monthly income of 2001–5000 predominate. In terms of the source of tourists, 70% of the tourists were from within Shandong Province, and 30% were from outside the province. At the level of the number of visits, the majority of tourists were those who had visited the city once, accounting for 66%.

**TABLE 2:** Basic demographic characteristics of respondents (*N* = 233).

Category	Classification	Number of individuals	Percentage/percent
Sex	Male	129	53
	Female	104	47
Age	<18 years	14	6
	18–30 years	70	30
	31–45 years	100	43
	45–60 years	42	18
	61 and over	7	3
Academic qualifications	High school and below	103	44
	College and bachelor’s degree	116	50
	Postgraduate and above	14	6
Vocational	Student	42	18
	Government/institution personnel	70	30
	Corporate employees	75	32
	Self-employed	23	12
	Retirees	7	3
	Other	11	5
Monthly income	2,000 and below	42	18
	2,001–5,000 yuan	128	55
	5,001–8,000 yuan	47	20
	8,001 and above	16	7
Place of residence	Mountain province	163	70
	Outside Shandong province	70	30
Taishan Scenic Spot visits	Visited 1 time	154	66
	Visited 2–4 times	47	20
	Visited more than 5 times	32	14

### 4.2 Hypothesis testing

#### 4.2.1 Structural equation model test

**FIGURE 2:**
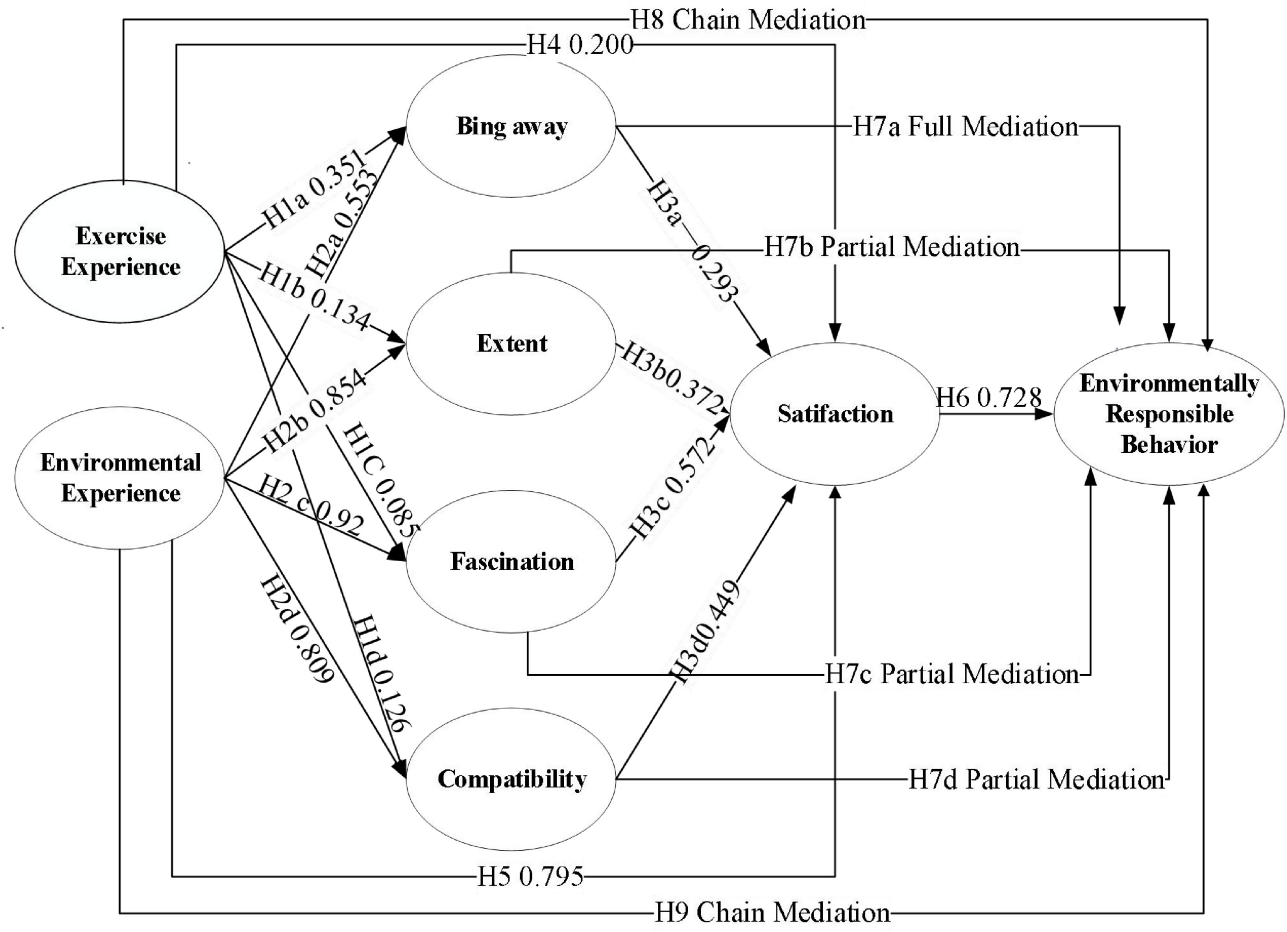
Path of standardized parameter estimation in the model.

With the help of Amos24.0 using the great likelihood method to test the model, the results are shown in the Figure 2 and Table 3, the tourists’ exercise experience positively affects the bing away dimension in the four dimensions of the tourists’ restorative environmental perception, and the hypothesis H1a is established.

**TABLE 3:** Results of structural model validation.

Research hypothesis	Path	Standardized path coefficient (β)	*t*-value	Standard error	Test result
H1a	Movement experience → bing away	0.351	3.317	0.122	Support
H1b	Motion experience →extent	0.134	1.642	0.064	Rejection
H1c	Movement experience → fascination	0.085	1.1115	0.060	Rejection
H1d	Motion experience → compatibility	0.126	1.378	0.067	Rejected
H2a	Environmental experience → bing away	0.553	5.582	0.143	Support
H2b	Environmental experience → extent	0.854	9.422	0.110	Support
H2c	Environmental experience → fascination	0.919	7.923	0.100	Support
H2d	Environment experience → compatibility	0.809	6.867	0.111	Support
H3a	Bing away → satisfaction	−0.293	−3.020	0.088	Rejection
H3b	Extended → satisfaction	0.372	1.894	0.303	Support
H3c	Fascination → satisfaction	−0.572	−0.963	0.441	Rejection
H3d	Compatibility → satisfaction	0.449	3.344	0.17	Support
H4	Motion experience → satisfaction	0.200	2.024	0.094	Support
H5	Environmental experience → satisfaction	0.795	1.263	0.781	Support
H6	Satisfaction → environmentally responsible behavior	0.728	1.504	0.045	upport

In terms of the influence of environmental experience on restorative perception, there is a significant positive influence of environmental experience on all four dimensions of restorative perception, with the overall performance of fascination (0.919) > extent (0.854) > compatibility (0.809) > distancing (0.553), which suggests that hypotheses H2a–H2d are supported; and in terms of the influence of restorative perception on satisfaction, there is a significant positive influence of the movement experience on the restorative environment. on satisfaction, both extent and compatibility have a significant positive effect on satisfaction, but farness and fascination do not have a significant effect on satisfaction, suggesting that hypotheses H3b and H3d are supported; tourists’ exercise experience and environmental experience both have a significant positive effect on satisfaction, and the extent of the effect of environmental experience on satisfaction is stronger than that of the exercise experience, suggesting that hypotheses H4 and H5 are supported; there is a significant positive effect of tourists’ satisfaction on the environmentally responsible behaviors significant positive influence, hypothesis H6 is supported.

#### 4.2.2 Mediation effect test

This study is based on the mediation effect test model proposed by Wen and Ye (2014). The mediation effect test was conducted using the SPSS Process plug-in, and the mediation effect test was conducted using the Bootstrap method to analyze the mediation effect of restorative environmental perception, satisfaction in the exercise experience and environmental experience on environmentally responsible behaviors, in which the sample size of the Bootstrap was is set to 1,000 and the significance level of the confidence interval is set to 95%, which is significant when the confidence interval does not contain 0.

Analysis such as the results (Table 4) show that the direct effect interval of bing away → environmentally responsible behavior includes 0, but the total effect and indirect effect confidence intervals do not include 0. Therefore, satisfaction plays a fully mediation effect between bing away and environmentally responsible behavior, and hypothesis H7a is supported.

**TABLE 4:** Results of mediation effect test.

Effect	Path	Effect value	Standard error
Total effect	Bing away → environmentally responsible behavior	0.424	0.060
	Ductility → environmentally responsible behavior	0.634	0.064
	Fascination → environmentally responsible behavior	0.601	0.063
	Compatibility → environmentally responsible behavior	0.550	0.059
	Movement experience → environmentally responsible behavior	0.407	0.069
	Environmental experience → environmentally responsible behavior	0.538	0.062
Direct effect	Bing away→ environmentally responsible behavior	0.1	0.07
	Ductility → environmentally responsible behavior	0.293	0.105
	Fascination → environmentally responsible behavior	0.244	0.101
	Compatibility → environmentally responsible behavior	0.198	0.096
	Motion experience → environmentally responsible behavior	−0.019	0.074
	Environmental experience → environmentally responsible behavior	0.069	0.105
Indirect effect	Distance → Satisfaction → environmentally responsible behavior	0.324	0.621
	Ductility → Satisfaction → environmentally responsible behavior	0.341	0.089
	Fascination → Satisfaction → environmentally responsible behavior	0.357	0.093
	Compatibility → Satisfaction → environmentally responsible behavior	0.352	0.099
	Movement experience → bing away → satisfaction → environmentally responsible behaviour	0.164	0.058
	Movement experience→extent→satisfaction→environmentally responsible behavior	0.179	0.058
	Movement experience → fascination → satisfaction → environmentally responsible behavior	0.175	0.05
	Exercise experience → compatibility → satisfaction → environmentally responsible behavior	0.176	0.058
	Environmental experience → bing away → satisfaction → environmentally responsible behavior	0.083	0.044
	Environmental experience → extent → satisfaction → environmentally responsible behavior	0.109	0.06
	Environmental experience → fascination → satisfaction → environmentally responsible behavior	0.067	0.052
	Environmental experience → compatibility → satisfaction → environmentally responsible behavior	0.147	0.062

There is a mediation effect between extent, fascination, compatibility and environmentally responsible behavior in restorative environmental perception, and the confidence intervals for the total and direct effects of extent → environmentally responsible behavior, fascination → environmentally responsible behavior and compatibility → environmentally responsible behavior do not contain 0, and the confidence intervals for extent → satisfaction → environmentally responsible behavior, fascination → satisfaction → environmentally responsible behavior confidence intervals for compatibility → Satisfaction → Environmentally Responsible Behavior confidence intervals do not contain 0. It can be seen that satisfaction plays a partially mediation effect in the effects of extent and compatibility on environmentally responsible behavior, and hypotheses H7b-H7d are supported.

Movement experience plays a positive effect on environmentally responsible behavior through the four dimensions of restorative environmental perception and satisfaction, and the confidence intervals for both the total and direct effects of movement experience → environmentally responsible behavior do not include 0. And in terms of the indirect effects of movement experience on environmentally responsible behavior, the confidence intervals for movement experience → bing away → satisfaction → environmentally responsible behavior do not include 0, and the confidence intervals for movement experience → extent → satisfaction → environmentally responsible behavior confidence interval does not include 0, exercise experience → compatibility → satisfaction → environmentally responsible behavior confidence interval also does not include 0, exercise experience → charm → satisfaction → environmentally responsible behavior confidence interval does not include 0. Based on this result, it can be seen that all four dimensions in restorative environmental perception play a chain mediation effect in the effect of exercise experience on environmentally responsible behavior, and the hypothesis H8a–H8d is supported, i.e., tourists influence restorative environmental perception through the exercise experience of mountaineering, which indirectly affects tourists’ satisfaction and will ultimately have an impact on whether they engage in environmentally responsible behavior.

Environmental experience plays a positive influence on environmentally responsible behavior through the compatibility dimension of restorative environmental perception and satisfaction. In terms of environmental experience, only the confidence interval of environmental experience→compatibility→satisfaction→environmentally responsible behavior does not include 0, and the confidence intervals of the remaining three dimensions of restorative environmental perception include 0, suggesting that only compatibility and satisfaction in restorative environmental perception play a chain-mediated role in the influence of environmental experience on environmentally responsible behavior. influence of environmental experience on environmentally responsible behavior, and hypothesis H9d is supported.

## 5 Research conclusion and discussion

### 5.1 Discussion

This study aimed to explore how tourists’ exercise and environmental experiences in mountain scenic areas influence their environmentally responsible behavior through the mediating mechanisms of perceived restorativeness and satisfaction. The empirical results not only validate most of the hypotheses but also reveal several nuanced and important mechanisms, providing new insights for both theory and practice.

#### 5.1.1 Theoretical implications

The primary theoretical contribution of this study lies in integrating and testing a sequential mediation model based on Attention Restoration Theory, which coherently links sports tourism experiences, psychological restoration processes, and pro-environmental behavioral outcomes. First, the finding that environmental experience had a strong and comprehensive effect on all four dimensions of perceived restorativeness, while exercise experience only significantly influenced “being away,” deepens our understanding of the differential “restorative potential” of various tourism experiences. The inherent quality of the natural environment serves as the cornerstone for triggering comprehensive restorative perceptions, whereas physical activity primarily provides a psychological gateway for “getting away” from daily routines. This echoes and extends the work of Zhang and Liu (2021), emphasizing the necessity to distinguish the unique psychological pathways of different experiences in composite settings.

Second, the study uncovered an interesting boundary condition regarding the differential impacts of restorative dimensions on satisfaction. Only “extent” and “compatibility” significantly enhanced satisfaction, while “being away” and “fascination” did not. This result partially supports findings from cultural heritage sites (Cho and Um, 2016) but offers a more refined explanation. In popular and often crowded destinations like Mount Tai, the sense of “being away” may be compromised by congestion, and “fascination” may not translate into satisfaction if it does not align well with personal expectations or environmental fit (Yang and He, 2016). This suggests that the translation of perceived restorativeness into satisfaction is not automatic and may be moderated by contextual factors (e.g., crowding) and person-environment fit, pointing to a valuable direction for future research.

Finally, the validation of the chain mediation effect, particularly that exercise experience influences environmentally responsible behavior entirely through the sequential path of “perceived restorativeness → satisfaction,” holds significant theoretical value. It illuminates the complex “psychological conversion chain” from physical activity to pro-environmental action: exercise first promotes psychological restoration (especially “being away”), the enhanced restorative experience then boosts visit satisfaction, and this positive affective state ultimately becomes the key driver of environmentally responsible behavior. The clarification of this pathway builds upon and refines previous research by moving beyond a focus on direct effects or single mediators, thereby offering a more integrated view of how tourism experiences translate into sustainable behaviors.

#### 5.1.2 Practical implications

The findings offer direct and actionable insights for the sustainable management of mountain scenic spots. First, management strategies should be differentiated. Scenic areas should prioritize ensuring and enhancing ecological environment quality (e.g., resource conservation, cleanliness), as this is the most effective way to strengthen tourists’ restorative perceptions and satisfaction. Concurrently, sports tourism programs should be carefully designed to emphasize safety, enjoyment, and social interaction to amplify their unique value in providing “being away.”

Second, scenic area planning should focus on enhancing perceptions of “extent” and “compatibility.” This involves creating coherent and rich landscape sequences and ensuring that facilities and services are highly compatible with the diverse needs of visitors (e.g., rest, exploration, photography). Optimizing tour routes, providing clear signage, and ensuring adequate amenities can significantly increase visitor satisfaction, thereby promoting environmentally responsible behavior.

Third, restorative experiences should be explicitly used as a core message in marketing and education. Through promotional materials and interpretive systems, scenic areas can guide tourists to consciously notice and appreciate the restorative qualities of the environment and explicitly link this positive experience to the behavioral expectation of “being a responsible tourist,” thereby motivating environmental commitment at an affective level.

#### 5.1.3 Limitations and future research

This study has limitations that indicate directions for future research. First, the data were collected from a single site (Mount Tai) using a cross-sectional design, which limits the generalizability of the findings and the strength of causal inference. Future research could replicate and test the model in different cultural contexts and various types of mountain destinations, employing longitudinal or experimental designs. Second, the measurements of exercise and environmental experiences were relatively general. Future studies could employ more granular measurements to uncover more micro-level mechanisms. Finally, relying mainly on self-reported scales, future research could incorporate physiological indicators to objectively measure restoration, or use behavioral observation to record actual environmentally responsible behavior, making the conclusions more robust.

### 5.2 Conclusion

Grounded in Attention Restoration Theory, this study constructed and validated a theoretical model integrating exercise experience, environmental experience, perceived restorativeness, satisfaction, and environmentally responsible behavior. Based on an empirical survey of tourists at Mount Tai Scenic Area, the main conclusions are as follows:

Both exercise experience and environmental experience are significant antecedents of tourists’ perceived restorativeness, with environmental experience having a more comprehensive and stronger effect.

The “extent” and “compatibility” dimensions of perceived restorativeness, along with both types of experience, significantly enhance tourist satisfaction.

Satisfaction plays a key mediating role between perceived restorativeness and environmentally responsible behavior. Furthermore, perceived restorativeness and satisfaction together form a significant chain mediation path through which exercise experience influences environmentally responsible behavior.

In summary, enhancing tourists’ exercise and environmental experiences in mountain scenic areas, and optimizing design to strengthen their perceived restorativeness (particularly extent and compatibility), represent effective pathways for promoting tourist environmentally responsible behavior and achieving sustainable destination development through the mechanism of satisfaction.

Notably, the findings highlight the multidimensional and asymmetric nature of restorative perception, suggesting that future research and practice should account for the distinct pathways through which different restorative dimensions operate.

## Data Availability

The original contributions presented in this study are included in this article/supplementary material, further inquiries can be directed to the corresponding author.
